# *Morzeddhu*: A Unique Example of a Traditional and Sustainable Typical Dish from Catanzaro

**DOI:** 10.3390/foods13121810

**Published:** 2024-06-08

**Authors:** Stefano Alcaro, Roberta Rocca, Maria Grazia Rotundo, Francesco Bianco, Luigi Scordamaglia

**Affiliations:** 1 Dipartimento di Scienze della Salute, Università “Magna Græcia” di Catanzaro, Viale Europa, 88100 Catanzaro, Italy; rocca@unicz.it; 2 Net4Science SRL, Università “Magna Græcia” di Catanzaro, Viale Europa, 88100 Catanzaro, Italy; 3 Associazione CRISEA, Centro di Ricerca e Servizi Avanzati per l’Innovazione Rurale, Loc. Condoleo, 88055 Belcastro, Italy; agronoma.rotundo@libero.it; 4 Antica Congrega Tre Colli, Viale De Filippis, 320, 88100 Catanzaro, Italy; francescobianco02@hotmail.com (F.B.);; 5 Fondazione Filiera Italia per la Distintività del Cibo, del Sistema Agroalimentare e della Trasformazione, Via Ventiquattro Maggio 43, 00184 Roma, Italy

**Keywords:** tripe, vegetables, nutritional value, mediterranean diet, fifth quarter, antioxidant properties

## Abstract

“*Morzeddhu*” in the local dialect of Catanzaro (“*Morzello*” in Italian) is an official typical dish of the capital of the Calabria region. It is a peasant dish, almost unknown at an international level, that labels, in an extraordinary way, the culinary identity of Catanzaro, a city founded around the X century. After America’s discovery, its preparation was optimized and definitively fixed. Its recipe is strictly based on a cow’s “fifth quarter” combined with spicy and typical Mediterranean vegetables. Remarkably, no pork meat is used, and when all traditional ingredients are included in the complex and quite long preparation of this special dish, it can deserve the title of “*Illustrissimo*”. This review provides a scientific description of Illustrissimo, emphasizing its unique properties and connection to the circular economy, food security, and the Mediterranean diet. We also highlight its unique quality compared to other alternatives through an analysis of their nutritional facts and bioactive compounds. Nutritionally, offal and fifth quarter components are a rich source of high-quality protein, with lower levels of total fat and saturated fatty acids compared to other meat cuts. In essence, this dish offers a great example of a high-quality yet affordable meal, aligning perfectly with a Mediterranean diet.

## 1. Introduction

### 1.1. Morzeddhu: A Typical and Sustainable Dish from Catanzaro

Catanzaro is the capital of Calabria, a southern region of the Italian peninsula neighboring to the north Basilicata and surrounded by two Mediterranean seas (the Tyrrhenian Sea to the west and the Jonian Sea to the east). According to an ancient mythological story, the term Italia could be used to refer to this area by the Greek king Italo, as reported by Aristotle [1].

In this context, several centuries ago, the citizens of Catanzaro developed a unique dish, mostly based on often wasted parts of ruminants, which included many flavors and tastes belonging to a Mediterranean diet [2,3,4,5,6]. It has never been reported at an international level in scientific publications and deserves to be studied thoroughly, starting from a sustainability point of view and some other nutrition and nutraceutical aspects. This review could be the starting point of a novel research line for the valorization of a typical product that risks disappearing in the coming decades. It can be linked to the EAT–Lancet Commission, which focuses on promoting healthy diets from sustainable food systems [7]. The commission recommends a shift from our current unsustainable eating habits to improve health and reduce environmental harm. To this end, the commission has recommended a transition towards plant-based food sources, prioritizing the consumption of locally and seasonally available products and minimizing the consumption of highly processed foods. The World Health Organization (WHO) defines sustainable, healthy diets as “eating patterns that promote optimal health and well-being with minimal environmental impact and pressure. These diets should be accessible, equitable, affordable, and culturally acceptable” [8]. While there is still uncertainty surrounding the exact components of a healthy and sustainable diet, some measurable aspects have been identified in the literature. These include (1) reducing excessive consumption, (2) increasing the intake of plant-based foods such as fruits, vegetables, grains, and legumes, (3) reducing animal-derived and processed foods, (4) focusing on local and seasonal products, and (5) minimizing food waste [7].

The main objective of this work is to explore the culinary, cultural, and nutritional aspects of *Morzeddhu*, a traditional dish from Catanzaro. This study aims to highlight the dish’s historical and cultural significance and its use of economical ruminant parts often overlooked in modern cuisine. This makes *Morzeddhu* a strong advocate for responsible food consumption, as it is both sustainable and eco-friendly. Additionally, this study examines the nutritional benefits associated with *Morzeddhu*. Our primary goal is to showcase the unique qualities of this dish, especially in comparison to other options or more expensive cuts of meat. *Morzeddhu* aligns perfectly with the principles of a Mediterranean diet, offering substantial nutritional value and a delicious taste. Ultimately, it is an exceptional example of a traditional meal that does not compromise on quality thanks to its cooking method, which effectively eliminates harmful microorganisms.

### 1.2. The Fifth Quarter Sustainability Problem: European Community Regulatory References

Food production impacts the environment differently, depending on the nature and type of food [9,10]. This phenomenon involves the problem of environmental sustainability, which has been particularly debated for some time. The diffusion and consumption of the fifth quarter of veal, the basic and prevalent ingredient of the typical Catanzaro dish “*Morzeddhu*”, historically peculiar to the less well-off social classes, reveals, in truth, a virtuous gastronomy—c. d. “circular kitchen”—where everything is used. It represents a new way of conceiving gastronomy, satisfying a taste that has its roots in a distant past. It must be said that offal, which forms the fifth quarter, is generally underestimated by the modern consumer and, consequently, by large-scale distribution, which prefers the “noble” parts to satisfy demand. This consequently determines the increase in waste related to slaughter, i.e., the waste and the lack of sustainability in terms of environmental impact caused by breeding activity. The empirical data show that using primarily “noble” parts of veal, such as fillet or sirloin, on a large scale increases waste material. Therefore, a greater and more conscious use of offal, following diverse traditional recipes from various regions, allows for the optimization of resources based on a given quantity. There is no doubt that the production of meat, aimed at specific consumption and restricted to some parts of the animal, has, as its consequence, forced the choice of intensive farming, which constitutes the real crux of the matter. The problem of food waste is a problem of quantity, so we should first make the waste bin slimmer before ourselves. Food that becomes food waste becomes an additional cost because it must be disposed of. In essence, we have an economic and ecological cost because it pollutes. According to the FAO, as early as 2009, 70% of people in poverty depended on intensive farming. According to a WWF report [11] within the Food4Future campaign, intensive farming causes 14.5% of total greenhouse gas emissions and uses 40% of cultivated land for the production of feed. This constitutes a global system that is seriously affected by the type of food consumed, as well as the way it is produced and transported. Now, beyond a preference for foods of plant origin, desirable in a vision that takes into account a healthy Mediterranean diet, the probable way out is given by a choice of strong rationality and awareness, which is not believed to be rigidly associated with the almost elimination of meat consumption in general but with its considered consumption. The solution to the problem should be closely linked to the promotion of more aware, healthy, and sustainable food systems. From this, taking into account the fact that the meat industry normally uses more resources than the production of plant foods, it would be necessary to favor some production methods which are undoubtedly more sustainable, such as pasture farming. A virtuous example is performed by the Swiss agricultural company Favre, a medium-sized company (about 25 hectares), which, with a view to preserving the climate and the environment, feeds the cattle with a particular mixture based on grass, hay, and concentrated feed, which allows for a drastic reduction in methane emissions. The latter element is produced by the digestive process of the aforementioned cattle. This is an example of targeted breeding, with the use of fodder combined with some food additives, which allows methane production by livestock to be reduced [12].

In this regard, the Italian breeding model is particularly virtuous too. In fact, based on the Ispra report of 2023 [13], emissions linked to livestock farming in Italy are equal to approximately 5% of the total amount and therefore equal to almost one-third of those at a global level. To produce 1 kg of meat in Italy (and generally in Europe), the emissions are equal to approximately 20% [8] of those emitted on the American or Asian continent (this is also linked to the high use of livestock waste, including the ruminal content obtained from emptying the tripe for the production of biogas and biomethane and therefore compost and digestate, an excellent natural fertilizer). Greater sustainability in the consumption of this dish can therefore be guaranteed by starting exclusively with animals raised in Italy.

From a regulatory point of view, it should be noted that last year the European Parliament approved amendments to the proposal for a Directive of the European Parliament and of the Council, which modifies the previous directive of the European Parliament and of the Council [14] relating to industrial emissions, excluding cattle farms from the regulations on industrial emissions. In essence, the text rejects the European Commission’s proposal to expand the activities covered by the directive to cattle herds of more than 150 animals. Recently, the Council and the European Parliament reached a provisional political agreement [15] on the revision of the Industrial Emissions Directive (IED). The agreement, provisional because it awaits formal adoption by both institutions, establishes certain “thresholds” for farms: 350 LU (adult livestock units) for pigs, 280 for poultry, and 380 for mixed farms, with the exclusion of extensive farms and livestock farming for domestic use from the scope of the next directive on industrial emissions, the main EU regulatory instrument regulating pollution caused by industrial plants and livestock farms for intensive farming.

### 1.3. Potential Nutraceutical Aspects and Perspectives

In the past, bovine tripe and offal were disregarded and considered to have little food value. The main drawback regarding the ingredients of *Morzeddhu* is concern about the risk of contamination with disease agents, including bacteria, parasites, viruses, and prions, due to the presence of tripe [16]. The consumption of slightly cooked offal dishes may cause foodborne illnesses. A famous example of this kind of disease is notably highlighted by the outbreak of “mad cow disease” (bovine spongiform encephalopathy, BSE). BSE, a neurological disorder in cattle caused by misfolded prion proteins, is transmissible to humans, leading to Creutzfeldt–Jakob disease (CJD). Specifically, it is theorized that these conditions arise from animals consuming feed previously contaminated with a prion agent, which accumulates in specific tissues like the brain, eyes, spinal cord, skull, vertebral column, tonsils, and distal ileum [17], none of which are included in the *Morzeddhu* meat selection. However, the risk of contamination by enterobacteria, such as *Salmonella enterica serovar*, *Campylobacter* spp., *Escherichia coli*, and enterococci, from edible offal as well as muscle meats could be abolished by preparing organs including liver and tripe at a minimum internal temperature of 160 °F (71.1 °C) [18]. This concern, alongside issues like high levels of phospholipids and cholesterol in some meat byproducts and the accumulation of heavy metals like cadmium in organs such as the liver and kidney, could represent some limits to its diffusion [19,20]. Anyway, the debate regarding the role of animal lipids and cholesterol in cardiovascular diseases and other health conditions persists due to contradictory evidence [21]. The liver and kidneys are crucial for monitoring metal contamination in animals, with heavy metals like cadmium posing potential risks to humans [20]. Techniques like supercritical fluid extraction can reduce cholesterol in cholesterol-rich meat coproducts without affecting other nutrients [22]. Furthermore, patents exist for removing heavy metals from food using specific ligands, offering potential solutions for heavy metal contamination in meat coproducts [23].

Nevertheless, it is worth noting that dishes featuring tripe have a long history in the cuisines of various nations, including Poland, Czechia, Austria, France, Germany, and across Asia [24,25,26,27]. These dishes often differ from their Italian counterparts in seasoning and preparation methods. Tripe, once cleaned, cooked, and sliced thinly, serves as a fundamental ingredient in vegetable soup, typically complemented by beef or smoked meat. It is crucial to cook the tripe in the soup until it reaches a soft texture. The soup is commonly served with a spicy flavor profile, featuring ingredients such as pepper, nutmeg, marjoram, ginger, and ground pepper. Preparation techniques may vary slightly based on regional and culinary preferences; for example, some may add a touch of tomato paste, while others forego the use of a roux. In Czechia, “dršťková polévka” enjoys popularity, distinguished by its characteristic red color from the significant inclusion of ground red pepper and cumin. In French cuisine, tripe from Caen, cut into squares and simmered with cider and calvados, holds a special place. Turkish cuisine boasts “işkembe çorbasi,” which is tripe seasoned with lemon, vinegar, and garlic and considered a national dish [28]. In Bulgaria, “shkembe chorba” stands out, differing from the Turkish variant mainly due to the addition of sweet milk [27]. In Latin America, “sopa de mondongo,” crafted from tripe and vegetables, enjoys popularity, though specific references would bolster this claim [29,30,31].

Interestingly, bovine tripe and offal have recently been re-evaluated due to their culinary and gastronomic importance and their nutritional benefits. These parts of the animal provide unique and rich flavors to dishes, and they are also sources of strategic molecules that are vital for the nourishment of the human body. Zinc, selenium, calcium, and chromium are some of the essential elements present in them, which are important for the proper function of various organs and tissues [32,33,34]. Therefore, the rediscovery of these parts of the animal is not only exciting for food fans but also for those who seek a balanced and nutritious diet. The correlation between nutraceuticals, health, cooking, and gastronomy has been re-established, as they work in conjunction to enhance the overall functioning of the human body. Such molecules can create a biological integration of nutrition by supplementing inorganic components, so everything ties in with the concept of functional foods. In recent times, there has been a surge of interest in them at an industrial level, which involves developing ingredients with additional health benefits beyond basic nutrition [35,36]. These foods are typically modified or enhanced with specific nutrients, vitamins, minerals, or bioactive compounds that can help manage or protect certain health conditions. For example, functional foods may include products like fortified cereals, probiotic yogurts, or omega-3-enriched eggs, which offer specific health benefits such as improved digestion, immune system support, or cardiovascular health [37]. The trend of using natural means to improve health and well-being has gained increasing popularity, leading to a significant milestone in the field of nutrition, with functional foods gaining considerable attention due to their potential health benefits [38]. As a result, there has been a growing interest in their research and development, recognizing a new promising area for innovation and investment in the food industry [39]. Overall, the emergence of functional foods is a positive development in the field of nutrition and has the potential to contribute significantly to public health [35]. In this regard, Catanzaro cuisine has unconsciously elevated the use of bovine tripe by incorporating various aromatic herbs that are being studied for their positive properties, including antioxidants. The question that arises spontaneously is, “Could *Morzeddhu* gain the definition of functional foods?” By analyzing specific aspects in this review, we can determine the importance of conducting further studies to accurately answer this question.

## 2. Brief History and the Preparation of the Dish

The tradition of *Morzeddhu* is quite old. The dominations that lived in Catanzaro in its history influenced strongly the development of typical dishes, as happened in many other places in Italy. In particular, the Arabian influence could explain the use of a bovine source instead of pork in its preparation. It is worth noting that the “selected pieces” for traditional *Morzeddhu* were extracted from the fifth quarter (*quinto quarto*), clearly revealing that this dish was formulated as a poor one, likely diffused among the lower class of citizens. In this regard, an ancient legend tells about a young woman, *Donna Chicchina*, who lived in a very poor district of the city. She was a widow with two children, and during the Christmas period, in order to bring something cheap but nutritionally consistent to her family, she used to clean the courtyards of Catanzaro notables by recycling the discarded pieces of beef. This should explain why no meats, or very limited parts, were included in her recipe based essentially on offal as the animal component. Along with this first ingredient, some others, selected again among inexpensive local vegetables typically belonging to a Mediterranean diet, were adopted, obviously taking into account the availability of them. After cooking all the ingredients for a very long time, as reported in Section 2.1, she likely ended up with a special soup that made not only her family but many other consumers in Catanzaro happy. According to a well-known tradition, this special dish used to be prepared very early in the morning, typically in taverns exclusively dedicated to it called “*putiche*”, where it was served from 9 to 11 am almost every morning, with some exceptions reported in Section 2.3. The typical consumers of this dish in the morning were actually laborers, who, after working hard in the early hours of the day, came to Catanzaro to deliver their products and then had a breakfast/lunch that refreshed them well. For sure, this tradition was typically carried out in the nineteenth century or perhaps even earlier, involving the “*putiche*” as a preferred location. The consumption of such a unique dish was strongly dependent on the availability of another culinary hero of the cuisine in Catanzaro, i.e., the special bread called “*Pitta*”, whose recipe is reported in Section 2.2. Its round, flat appearance and its typical rheological properties are strictly related to the multiple leavening techniques used to correctly prepare it. The way used to combine them is also quite unique because the innkeeper used to ask the restaurant guests if they preferred *Morzeddhu* within the *Pitta* bread or separated, i.e., on a soup plate. This gentle query for thoroughbred citizens of Catanzaro was a “rhetorical” one because the former solution is the traditional one but makes it more complicated to eat. Actually, the direct contact of the hot and juicy meat soup with the bread induces the need to consume the dish quite quickly, which would otherwise cause the bread to fall apart in a very few minutes. People from Catanzaro know this side effect quite well and how to prevent other negative consequences too. Before going on with the details about the preparations of each component and other variations, it is important to explain who decided to label the combination of *Morzeddhu* and *Pitta* and why. The local poet Giovanni Sinatora (Borgia, CZ, 1877–1937) was the first to draw attention to this happy mixture, dedicating to it the adjective “*Illustrissimo*” (most illustrious) in rhymes in the early years of the nineteenth century. Many years later, another author, Enzo Zimatore (Catanzaro, 1908–1991), a well-known president of the Council of the Lowers’ Association of Catanzaro, dedicated a short volume to this special dish, reproposing the same label [40]. Then, several other authors completed the description, often using the local dialect to celebrate it.

### 2.1. Preparation of the Morzeddhu

The components of the dish can be distinguished into two main parts: the ruminant and the vegetable ones. Another minor one is related to the inorganic components. Recently, the official recipe of the dish, with exact quantities of each ingredient, was defined as reported in Table 1.

Tripe is the main ruminant component of the dish, i.e., based on the four stomachs of beef: rumen, reticulum, omasum (*centupezzi*), and abomasum (*quagghiaru*). Other fifth quarter components are part of the traditional recipe of *Morzeddhu*: the lungs, the intestines (large and caecum), cow fat (kidney or heart), sweetbreads (glands), the esophagus, the trachea, and the heart (optional upper part, so-called “delicate”). The spleen is also included but must be added at the end of the preparation. All ruminant components can be considered the most sustainable section due to their normal exclusion in most food preparations.

The second part is related to some typical Mediterranean diet vegetables, listed as follows: tomato concentrate, hot peppers, pepper concentrate, laurel, and oregano.

Washing and cutting stages

**Figure 1:**
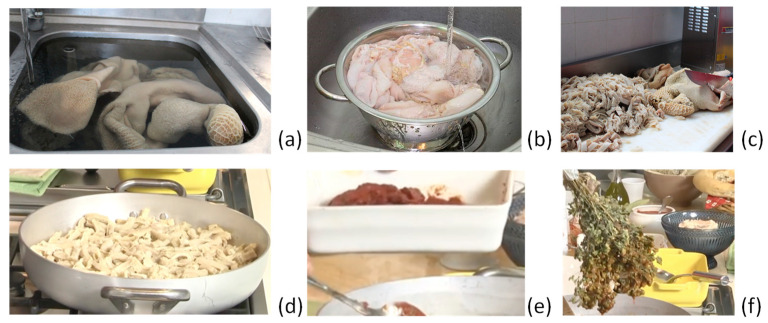
Main steps in the traditional preparation of *Morzeddhu*: washing (**a**), rinsing (**b**), cutting (**c**), boiling and sautéing (**d**), adding the spleen (**e**), and mixing with oregano (**f**).

The preparation starts with a careful washing of the tripe of all the innards (Figure 1a). Rinse them with no chemical additives. Only lemon juice and some hard work are suggested. After washing, leave the offal to soak in salted water with lemon for a few hours (about two), rinsing it thoroughly afterwards (Figure 1b). Then, the cutting of all of the fifth quarter is crucial. Usually, the average of the maximum suggested dimensions of each piece should not exceed 3 cm in length and 1 cm in thickness and width (Figure 1c).

Cooking offal and aromas

It is necessary to boil the tripe, the other offal, and the spleen separately for about 20 min, frequently changing the water (Figure 1d). Sauté the tripe using only the fats of the ruminant part, *pepperoncino*, and dry laurel, and add the concentrates with the other offal, except for the spleen, to avoid darkening (Figure 1e). Leave to cook for 4 h. Use the branch of oregano to mix the soup, always keeping it hot, and add the spleen near the end of the preparation (Figure 1f). At the end, the row of offal components, such as the trachea, should be removed from the soup, obtaining a red, dense, and tasty sauce. Some laurel leaves should be added at the end.

**Figure 2:**
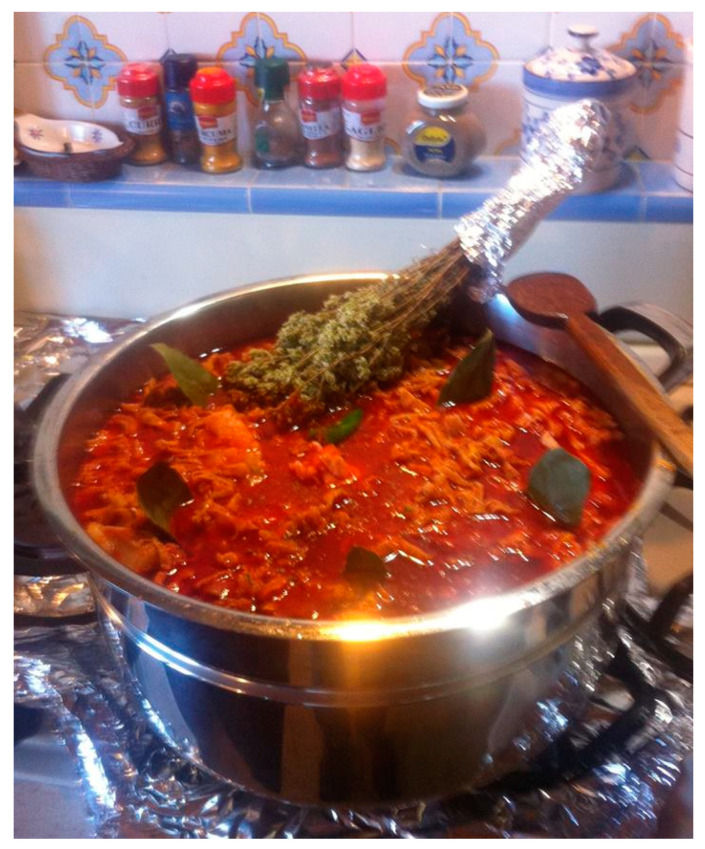
Final look at the *Morzeddhu*.

The consistency of the *Morzeddhu* is that of a dense, hot soup (Figure 2). The aroma of laurel and oregano is strong. This last vegetable, usually as a dry branch, is often adopted as a mixing ladle, also allowing the release of its components, which will be discussed in Section 3. According to some experts, the term *Morzeddhu* could be connected to the small size of the carefully cut offal, and, based on others, it concerns the method of consumption: biting (*a morzi*) for its version served in *Pitta*.

### 2.2. Preparation of the Pitta

This traditional bread from Catanzaro is unique for some reasons: (a) its shape looks like a bicycle wheel; (b) its preparation is based on multiple leavening processes, thus conferring a unique softness; and (c) it is served open like a sandwich, hosting the hot *Morzeddhu* soup. Therefore, it quickly starts to adsorb it, prompting the consumer to eat it as soon as possible. The ingredients of this bread are very simple: type 0 unrefined flour (500 g), salt, warm water (350 g), and natural yeast (12 g).

**Figure 3:**
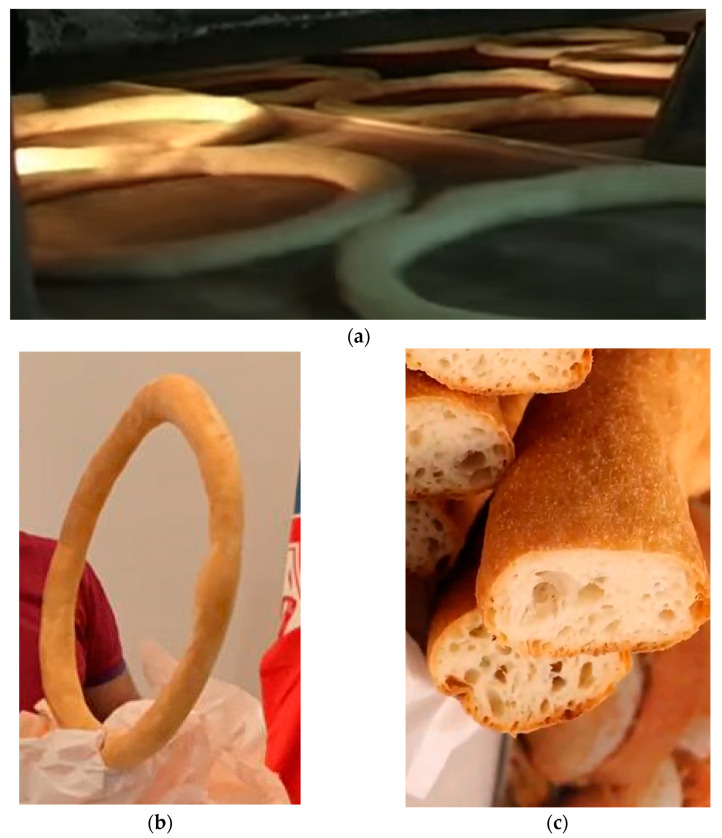
The *Pitta* bread from Catanzaro: oven cooking (**a**), size and aspect (**b**), and cut section (**c**).

Everything is mixed, putting the salt in at the end (to allow for the gluten network), and it is left to rise (first leavening) for about 45 min—leavening the mass. Then, we proceed with weighing and rounding; it is left to rest (second leavening) for about 20/25 min, after which it is pierced and a tight ring is formed, and then it is left to rest (third leavening) for about 15/20 min. The ring is subsequently enlarged to form a diameter of about 45/50 cm, with a width (thickness/ring section) of about 3/4 cm. Then, it is left to rest again on special frames (fourth leavening) for up to about 15 min. Finally, it is placed in the oven for 15/18 min at a temperature of 220/230 °C (Figure 3a). The *Pitta* must be strictly matte, not shiny (Figure 3b), and soft (Figure 3c).

### 2.3. The Marriage of Traditional Morzeddhu and Pitta

**Figure 4:**
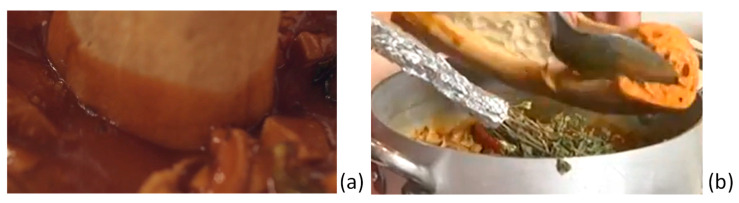
Assembling the *Illustrissimo*: wetting the extremities of the *Pitta* (**a**) and filling the open *Pitta* with the *Morzeddhu* (**b**).

As already mentioned, some experts on this dish have already labeled the combination of the hot, spicy soup and the special bread from Catanzaro as “*Illustrissimo*”. There is a technique for quickly preparing this “marriage” to let the consumer enjoy it in the best way. Basically, it is important that the *Pitta*, cut from the wheel to obtain about four/five portions, be opened like a book to host the *Morzeddhu* in a semi-arched envelope. Before filling it, another precaution should be paid. The two extremities of the Pitta portion should be inserted in the soup to wet them with the hot sauce (Figure 4a). This procedure ensures that any part of the bread, including the extremities, can correctly adsorb it. Then, the open bread can be filled with the soup, trying as much as possible to include all the offal components, easily detectable by their aspect and consistency (Figure 4b). The spleen and heart are always darker than other tripe components.

**Figure 5:**
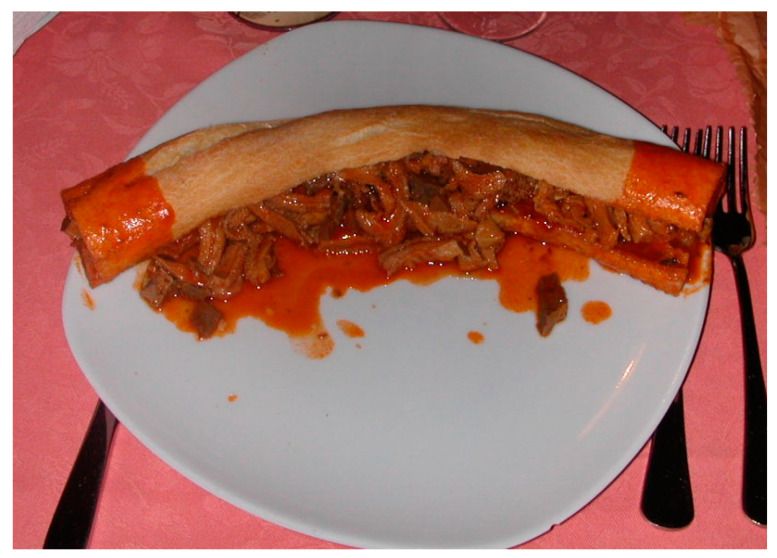
The *Illustrissimo* ready to be served.

Then, the *Illustrissimo* is ready to be served on a simple plate and eaten, usually starting from one extremity (Figure 5).

### 2.4. Other Morzeddhu Recipes

There are other varieties of *Morzeddhu* that should be cited. They cannot be considered *Illustrissimo*, even if combined with the *Pitta*.

**Figure 6:**
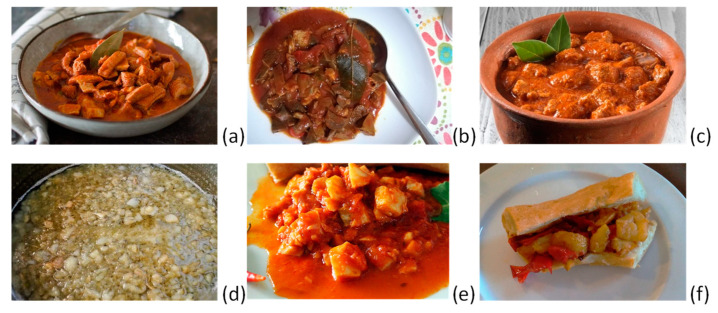
Other varieties of Morzeddhu: (**a**) the “light” version of Morzeddhu; (**b**) minute; (**c**) soffritto; (**d**) cicoli; (**e**) baccalà; and (**f**) MorzelloRey.

(a) A “light” version of the *Morzeddhu* (Figure 6a) is that where the ruminant components are limited only to the tripe, i.e., skipping the spleen, lung, heart, intestine, cow’s fat, sweetbreads, esophagus, and trachea. Clearly, this version lacks a lot of the strong taste of the traditional recipe, but often, it is justified by the difficulty of obtaining all the other components at a butcher’s.
(b) Another special version, really for refined palates, is that based on the ruminant and offal components of suckling lamb (in dialect *minuto*) (Figure 6b). This version is quite hard to obtain due to difficulties in acquiring the young animals during the year, but it is tastier than the traditional one due to the particular wild flavor of the ovine.
(c) A more diffused version, reminiscent of the traditional one for most of the Southern Italian region, is based on pork instead of beef. In this case, the meat is used as well as wine for deglazing the preparation. Its name, *soffritto*, is literally translated from the term “sautéed” (Figure 6c). The taste is different, but again, it should be served with the *Pitta* bread in Catanzaro.
(d) A quite caloric and heavy version of *Morzeddhu*, mostly eaten in winter, is based on fat portions of pork, corresponding to greaves (*cicoli*), which replace all the ruminant components (Figure 6d). This is a white version of the *Morzeddhu* because the vegetable ingredients are also not included, with the only exception being the crushed *pepperoncino*, which should be sprinkled on after filling the *Pitta* with the *cicoli*. Only a small portion of this ultratasty *Morzeddhu* is recommended in order to limit the cholesterol intake.
(e) In order to make possible the consumption of *Morzeddhu* every day of the week, citizens of Catanzaro invented a fish version where all the ruminant parts are replaced with salted cod (*baccalà*) (Figure 6e). The recipe is much faster to make than the traditional one because this fish is cooked quickly. The sauté involves the use of extra-virgin olive oil. This version is the official one for special days of Catholic tradition, such as Saint Friday before Easter and other occasions when the consumption of meat should be forbidden.
(f) The latest version of *Morzeddhu* was proposed in 2023 by the Antica Congrega Tre Colli di Catanzaro. It is based on an innovative recipe inspired by another tradition of the city. This is the only version that contains neither meat nor fish in its composition. Two traditional components of a Mediterranean diet, the yellow potato from the Sila and the red bull pepper from Calabria, are sautéed with only extra-virgin olive oil and salt to give rise to a classic side dish of the Calabrian cuisine known as “*patati e pipareddhi*”. Once cooked, they should be used to fill the *Pitta* bread in the same way as the other dishes. The yellow-red colors are important as they are reminiscent of the football team of Catanzaro, which in 2023 was actually performing very well. Since one of the heroes of the glorious soccer team was Massimo Palanca (called *oRey*), this special innovative traditional recipe of the vegetable *Morzeddhu* was called “*MorzelloRey*”, officially presented on 30 September 2023 and dedicated to him (Figure 6f).

## 3. Nutritional Facts and Bioactive Compounds of Traditional *Morzeddhu*

Traditional *Morzeddhu*, i.e., that definable as *Illustrissimo* if combined with *Pitta* bread, is a traditional dish from Catanzaro based on beef offal and other vegetables.

Biel et al. conducted a study to compare the micro- and macro-nutrient content of meat components, including the liver, heart, kidneys, tongue, and brain, with *musculus semitendinosus* in veal, beef, and lamb [41]. The study found that they are a rich source of inorganic elements such as zinc, iron, copper, manganese, calcium, and sodium. The amount of them in the meat components was significantly higher than in the muscular tissues [41]. According to a study by Lynch et al. [42], meat offal generally contains a higher vitamin content compared to lean meat. Fat-soluble vitamin A is particularly abundant in offal, with a higher proportion found in the liver and kidneys [19]. In addition, meat components are an excellent source of water-soluble vitamins B1 (thiamin), B2 (riboflavin), B3 (niacin), B5 (pantothenic acid), B6 (pyridoxine), B9 (folacin), B12 (cobalamin), and C [20]. Some studies have revealed that beef liver, heart, kidney, abomasum, and pancreas contain the highest vitamin B1 content compared to other coproducts, while the vitamin B2 content is higher in the liver and kidneys compared to pork, beef, veal, and muscle tissue from chicken and rabbits [43]. Furthermore, the liver is an excellent source of vitamin B3, B6, B9, and B12, ascorbic acid, and vitamin A, while the kidney is a very good source of vitamin B6, B12, and B9 [44]. These findings demonstrate the potential nutritional benefits of incorporating meat coproducts into the diet. Specifically, when examining bovine spleen and heart, which are prominent ingredients in Morzeddhu, it is important to note their rich nutritional profile with respect to tripe. These types of offal, analyzed by an official nutritional web resource [45], are known to contain a variety of vital components, including iron, phosphorus, potassium, selenium, and vitamin C. A comparison with the selected meat intended as muscle tissue and other kinds of beef, generally referred to as uniform in quality and normally leaner than higher-grade meat, could be carried out following a recent review by Soladoye et al. [46]. Meat coproducts are commonly considered a rich source of high-quality protein [47]. In particular, the protein content of the liver, spleen, and heart from beef carcasses is higher than that of other coproducts, including selected meat. Similarly, selected beef muscles contain a higher amount of total fat and saturated fatty acid compared to all other evaluated meat coproducts from beef [16,20,46,48,49,50]. A comparison of *Morzeddhu* with other kinds of beef or similar dishes is shown based on some preliminary data reported in Table 2. Specifically, we reported the main nutrition facts for the tripe bovine, which is the raw material of *Morzeddhu*, and for other similar dishes, including *Lampredotto* and *Gulash*. As shown in Table 2, despite the cooking process, *Morzeddhu* maintained the main nutrition factors of tripe bovine, with a reduced increase in energy value. Moreover, when compared to its counterparts, *Morzeddhu* has a distinct advantage in terms of its nutritional profile, with less energy and fat content.

Taking into account the preparation of the dish, it includes some other components different from the offal, such as hot peppers, laurel, pepper concentrate, and tomato concentrate, as reported in Section 2.1. These vegetables are frequently found to harbor pesticide residues, posing a significant threat to the quality of traditional dishes. It is essential to remain vigilant about this potential issue to uphold the excellence of our culinary creations. Specific attention must be paid to the risk of phytosanitary residues, especially regarding chili pepper and bay leaves, necessitating strict adherence to legislative standards. However, the dish might contain some bioactive compounds, mainly attributable to the following vegetable ingredients. The analysis is not based on analytical measurements carried out on *Morzeddhu* but only on qualitative considerations.

Hot pepper (*peperoncino*)

This spice is likely the most typical ingredient in Calabrian cuisine. Actually, it is an imported ingredient because it was introduced in Europe only after the discovery of the Americas, but in Calabria, it is so diffused that it can mostly be considered a determinant for many recipes and for *Morzeddhu* too. The main component responsible for the spicy effect is capsaicin [51]. Its presence in the dish is mandatory but can be quantitatively different on the basis of the preferences of the cooker and the type of *peperoncino* used. The most interesting compounds, also detectable in the dish, are related to the family of capsacinoids. Capsaicin (trans-8-methyl-N-vanillyl-6-nonenamide) is the most investigated alkaloid in this series. Recently, several biological properties were addressed in this compound, including its antimicrobial effects [52], anti-inflammatory and neuroprotection activities [53,54], and inhibition properties against two carbonic anhydrase isoforms, IX and XII, useful for preventing some neoplastic forms [55]. Its antioxidant profile is guaranteed by the phenolic moiety [56]. Another component with similar properties, well concentrated in *peperoncino*, is ascorbic acid [57].

Laurel

Laurel leaves are added at the end of the cooking process, so, even if their concentration is low with respect to the other vegetables included in the official recipe of the dish, their presence is evident just by smelling only the final product. Most of the scents deriving from these leaves can be attributed to the terpene compounds released when the hot soup hydrates and warms the dried laurel. Most of them, well represented in cultivated plants, are low-molecular-weight molecules that are quite volatile and aromatic, such as aromandrene, caryophyllene, thymol, linool, α-terpinol, α-terpinyl acetate, selinene, farnesene, and cadinene, while wild laurel is characterized by a consistent content of eugenol [58]. Most of them can contribute to the antioxidant protection of the dish.

Oregano

In the traditional preparation of *Morzeddhu,* a branch of oregano (about 50 g) is strongly suggested to be used as a ladle for the last part of the cooking procedure, i.e., when the soup is boiling to reduce the sauce. This process is often quite long and promotes the release of a lot of bioactive principles and flavors that complete the smell and taste of the dish. The compounds most represented in the edible part of the plant (flowers, leaves, dried or fresh) are aromatic compounds such as carvacrol, timolol, eugenol, and thymoquinone, characterized by low molecular weight and phenolic hydroxyl, conferring to them volatility and antioxidant properties [59,60,61].

Pepper concentrate

This component is likely the most peculiar from an organoleptic point of view because it is relatively uncommon in the preparation of other Mediterranean diet-based dishes. Pepper fruits are the source of the concentrate used for the preparation of *Morzeddhu*. There are several varieties of them that can be used for the preparation of the pepper concentrate. They are all rich in metabolites with potential health-promoting properties. Among them, the most relevant are ascorbic acid, carotenoids, flavonoids, and tocopherols [62]. Also, capsaicinoids are an ingredient, but the most relevant contribution of these derivatives comes from *peperoncino*. The overall antioxidant level of the dish is definitively attributable to this ingredient [63].

Tomato concentrate

The tomato concentrate is the most relevant vegetable component of the recipe. It is based on one of the most representative Mediterranean diet components. Tomato, as with other vegetables in this dish, entered Europe after the discovery of America but has been adopted in all of Italian cuisine. The most interesting bioactive compounds related to this ingredient are so-called carotenoids. The relevant molecules in this series are lycopene, phytoene, neurosoporene, and the famous carbocyclic derivative β-carotene. These secondary metabolites, typical of the tomato fruit, are products of terpene polymerization [64]. More recently, these derivatives were reported as potentially active against neoplastic diseases, acting through a multi-targeting mechanism of action [65].

## 4. Discussion

*Morzeddhu*, a peculiar dish of Catanzaro, boasts centuries-old traditions that are a consequence of the dominations that have passed through the city in its history. It is a poor plate based on very cheap ingredients. As concerns the ruminant parts, they are definitely waste products from cattle slaughter, normally unused in cuisine. They belong to the fifth quarter of the bovine, which has recently attracted the attention of many experts on traditional cuisine in Italy. Their combination with vegetables, following quite a long preparation, converts these minor ingredients into an extraordinary dish that has been appreciated in important forums such as the Salone del Gusto in Turin (2014 and 2022 editions) and the Expo in Milan (2015 edition). More recently, it has been discussed during the Italian National Food Chemistry meeting in Marsala [66], attracting the attention of a qualified scientific audience. Its “marriage” with the typical *Pitta* bread of Catanzaro completes the unique characteristics of this dish, making it deserving of the nickname “*Illustrissimo*”. Despite this, there is a concrete risk, especially among recent generations, of it not being prepared habitually, with the consequence that a traditional bastion of poor cuisine could be erased. Especially among young consumers in Italy, it is now common to quite often eat hamburgers, i.e., selected meat, at lunch or dinner, with potential negative health effects [67]. Looking at the nutritional facts reported in Table 2, there are some considerations that can be made by comparing the standard values of selected meats (Table 2). The first is that the caloric content of *Morzeddhu* is less than 50% with respect to beef. The fats contained in the same quantity of product are also less than 50%. The comparison of sugars reveals a slightly higher concentration (about 52%), but still much lower than the reference food. The carbohydrate contents are considerably lower (only 15%) than those detectable in selected meats. As expected, considering the vegetable contents of the *Morzeddhu* recipe, the protein intake is lower than 40%, while conversely, the ash total quantity is almost double that of the selected meat. Except for the protein contents, all the nutritional factors of *Morzeddhu* are attractive from a dietetic point of view. Moreover, the antioxidant defense that almost all vegetable components can release in the dish can also be interesting from a healthy point of view. Some of them are antioxidants able to exert protective biological effects through free-radical scavenging, protein binding, and interactions with human signal transduction pathways [59,60,65]. It is also interesting to note that some components of a Mediterranean diet, based on oregano, chili pepper, and other spices, have been highlighted as potentially valid supplements of bioactive agents useful in the prevention of metabolic syndrome diseases [68]. Obviously, these considerations are to be considered only preliminary because a full analysis of the food’s chemical characteristics should be carried out, starting with the ingredients used for the preparation of the dish.

The primary concern surrounding the ingredients in *Morzeddhu* pertains to the potential risk of contamination with disease-causing agents such as bacteria, parasites, viruses, and prions, given the presence of tripe [16]. However, this problem can be overcome, as reported above, by long cooking at a minimum temperature of 160 °F (71.1 °C) [18]. Despite this, it is stimulating to note how numerous studies have examined the relationship between offal consumption and its potential benefits in terms of protein, mineral, and fatty acid content, as well as the presence of essential nutrients such as selenium and zinc [69,70,71,72]. Interestingly, research has demonstrated a positive correlation between the consumption of meats such as tripe and *lampredotto* and a reduction in skin problems, mineral deficiencies, and melanoma risk. Interestingly, Yen et al. [73] have reported that regular consumption of red and processed meat could be associated with a decreased risk of melanoma. Further investigations are necessary to fully understand the relationship between offal consumption and its potential health benefits. Exploring the beneficial properties of *Morzeddhu* through the literature and studies remains an intriguing avenue. Understanding how the properties of raw and cooked tripe evolve due to cooking methods, temperature variations, cooking time, and the type of tripe used is an initial point of interest. It is noteworthy that the hygienic safety of the product in its raw state is a concern. The cooking process and its duration are pivotal factors in addressing this concern. Therefore, leveraging chemometrics in future research within the food industry holds promise for unraveling the origins and authenticity of traditional dishes like tripe or *Morzeddhu*. Undoubtedly, further studies are necessary to delve deeper into the advantageous qualities of *Morzeddhu*. As concerns the sustainability issue, it is clear that traditional *Morzeddhu*, as well as its variations based on the ruminant components, represents an interesting model to promote the recycling of a large part of the fifth quarter. The recipe for this dish could be promoted at a national and international level, linking it to the quality of the product and the traditional heritage that it represents for a city and a region still rich in ancient practices that cannot be lost in the name of food globality.

With respect to the quality control of this dish, a “National Certification Mark” has been recently deposited and registered at the Italian Patent and Trademark Office of the General Directorate for the Protection of Industrial Property—Ministry of Business and Made in Italy. The aforementioned Certification Mark (depicting a typical dish of Catanzaro cuisine with a symbolic reference to the “Morandi” Bridge in the city of Catanzaro) is the exclusive property of the no-profit cultural association “Antica Congrega Tre Colli” di Catanzaro. The deposit procedure was completed on 26 July 2023 (protocol nr. 302023000114684) and approved on 11 January 2024. The registration is valid for 10 years starting from the date of the deposit, and it is valid for the whole of Italy. Its application in the next few years will help to accurately track the production of the original *Morzeddhu* and likely help monitor the consumption of offal with respect to other bovine meats.

This topic and this dish should be the objects of additional studies in order to complete the profile of *Morzeddhu* from all food chemistry points of view. Offal, which was once viewed as waste material for the impoverished, has gained recognition due to its high nutritional value. Overall, these findings suggest that offal may be a valuable dietary component for individuals seeking to maintain a healthy, nutrient-rich diet.

## 5. Conclusions

In conclusion, *Morzeddhu*, a typical dish in Catanzaro, reflects the city’s rich historical and cultural heritage while utilizing inexpensive, often discarded, ruminant parts. Despite its potential risk of contamination, proper cooking methods can mitigate these concerns. It is worth noting that the careful selection of the starting material, especially the meat offal, and the accurate washing procedures indicated (Section 2.1) are essential steps for eliminating any risk of contamination. This dish offers nutritional benefits, including lower caloric content and fats compared to selected meats, along with notable antioxidant properties. Promoting *Morzeddhu* at national and international levels can aid in preserving traditional culinary practices and sustainable food production. The recent registration of a “National Certification Mark” underscores its cultural significance and ensures quality control. Further research is essential to fully explore *Morzeddhu’s* food chemistry profile, emphasizing its value as a nutrient-rich component of a healthy diet, considering the concept of nutraceuticals, introduced by Dr. Stephen De Felice in 1989 [74].

## Figures and Tables

**Table 1:** Ingredients and doses for the preparation of *Morzeddhu* for about 4 or 5 people.

Ingredients ^1^	Components	Quantity (g) ^1^
Tripe (abomasum, omasum, reticulum, and rumen)	Ruminant	1500
Spleen	Ruminant	200
Lung	Ruminant	200
Heart (upper part, so-called delicate)	Ruminant	300
Intestine (large and cecum)	Ruminant	200
Cow’s fat (kidney or heart)	Ruminant	150
Sweetbreads (salivary glands)	Ruminant	100
Esophagus	Ruminant	200
Trachea	Ruminant	100
Tomato concentrate	Vegetable	600
Pepper concentrate (two tablespoons)	Vegetable	10
Spicy chili pepper (one fresh *peperoncino*)	Vegetable	40
Oregano (one branch)	Vegetable	50
Laurel (4–5 dry leaves)	Vegetable	50
Salt	Inorganic	to taste
Water	Inorganic	5000

^1^ Official data from the 2nd *Morzeddhu* School organized by the Antica Congrega Tre Colli (November 2023).

**Table 2:** Comparison of main nutrition facts among *Morzeddhu*, selected beef meat, tripe bovine, *Lampredotto*, and *Gulash*. These data were determined considering 100 g of each product.

Nutritional Content	*Morzeddhu* ^1^	Selected Meat ^2^	Tripe Bovine ^3^	*Lampredotto* ^4^	*Gulash* ^5^
Energy value (cal/J)	110/461	250/1050	108/453	203/847	141/592
Fats (g)	4.81	10.00	5.00	11.00	6.3
Saturated fats (g)	1.51	5.20	-	3.6	2.7
Carbohydrates (g)	3.10	20.00	-	3.6	5.6
Sugars (g)	1.41	2.70	0	>3.6	2.1
Proteins (g)	13.56	35.00	15.8	22	15
Ash (g)	1.53	0.81	-	-	

^1^ Data as published by the Genius vendor of *Morzeddhu*. ^2^ Data as published by Soladoye et al. [27]. ^3^ Data as published by CREA [31]. ^4^ Data as published by Osteria Fumetti [32]. ^5^ Data as published by Società Agricola Maso dello Speck SRL [33].

## Data Availability

The data presented in Table 2 are available at the reference number [48]. No new data were created or analyzed in this study. Data sharing is not applicable to this article.
